# Chemical Composition and Antioxidant Profile of Sorghum (*Sorghum* *bicolor* (L.) Moench) and Pearl Millet (*Pennisetum* *glaucum* (L.) R.Br.) Grains Cultivated in the Far-North Region of Cameroon

**DOI:** 10.3390/foods11142026

**Published:** 2022-07-08

**Authors:** Saliou Mawouma, Nina Nicoleta Condurache, Mihaela Turturică, Oana Emilia Constantin, Constantin Croitoru, Gabriela Rapeanu

**Affiliations:** 1 Department of Biological Sciences, Faculty of Science, University of Maroua, Maroua P.O. Box 814, Cameroon; mawouma2001@yahoo.fr; 2 Faculty of Food Science and Engineering, Dunarea de Jos University of Galati, Domnească Street 111, 800201 Galati, Romania; nina.condurache@ugal.ro (N.N.C.); mihaela.turturica@ugal.ro (M.T.); oana.constantin@ugal.ro (O.E.C.); 3 Academy of Agricultural and Forestry Sciences, 61 Marasti Blvd., 011464 Bucharest, Romania; c.croitoru@sodinal.com

**Keywords:** sorghum, pearl millet, chemical composition, antioxidant activity, Far-North Cameroon

## Abstract

Sorghum and pearl millet are grain crops that can grow in semi-arid climates, with nutritional and bioactive properties superior to those of major cereals such as rice, wheat, and maize. However, these properties vary a lot, depending on the genetic factors, growing conditions, and place of cultivation. Four sorghum and two pearl millet grains cultivars grown in the Far-North region of Cameroon were screened for their chemical composition and antioxidant profile. The proximate and mineral analyses were performed using AOAC standard methods. The antioxidant profile was assayed spectrophotometrically and details on the phenolic compounds were investigated using HPLC. The pearl millet cultivars, especially *mouri*, showed higher contents of proteins, lipids, ash, calcium, copper, iron, and zinc. The *red* sorghum specifically exhibited the greatest amounts of total polyphenols (82.22 mg GAE/g DE), total flavonoids (23.82 mg CE/g DE), and total 3-deoxyanthocyanidin (9.06 mg/g DE). The most abundant phenolic compound was gallic acid, while the most frequent were chlorogenic and ferulic acids. The maximum antioxidant activity against DPPH was observed in *yellow-pale* sorghum (87.71%), followed by *red* sorghum (81.15%). Among the studied varieties of cereals, *mouri* pearl millet and *red* sorghum were the best sources of nutrients and bioactive compounds, respectively. Their consumption should be encouraged to tackle nutrient deficiencies and non-communicable diseases within local populations.

## 1. Introduction

Sorghum (*Sorghum bicolor* (L.) Moench) and pearl millet (*Pennisetum glaucum* (L.) R. Br.) are cereal crops belonging to the Poaceae family, which are native to Africa and were domesticated between 3000 and 5000 years ago [1]. After wheat, rice, maize, and barley, sorghum and pearl millet are the most widely produced grains on the planet [2,3]. Sorghum and pearl millet can endure a variety of environmental conditions, including low soil fertility, high temperatures, and insufficient rainfall. Sorghum and pearl millet are cultivated particularly in the semi-arid parts of Africa and Asia, where they are predominantly used for human consumption and are staple foods for the local populations [4,5,6]. In the Far-North region of Cameroon, as in other Sahelian parts of Africa, sorghum and pearl millet, which are consumed as gruel, rolled balls, partially cooked grains, and fermented beverages, are less expensive sources of nutrients for low-income individuals. They are nutritionally superior to the major cereals in protein, energy, vitamins, and minerals [7]. Additionally, sorghum and pearl millet are excellent sources of bioactive chemicals that help to improve non-communicable disease characteristics, such as obesity, diabetes, cardiovascular diseases, and cancer [8,9,10].

The pericarp of sorghum contains significant amounts of non-starch polysaccharides; phenolic chemicals such as 3-deoxyanthocyanidins, tannins, and phenolic acids; and carotenoids. The germ is composed of lipids, fat-soluble vitamins, B-complex vitamins, and minerals, whereas the endosperm is rich in carbohydrates, proteins, B-complex vitamins, and minerals [1].

Proteins, carbohydrates, dietary fiber, and minerals are all present in finger millet. Among all cereals, it contains the most calcium (344 mg/100 g). However, phytates, polyphenols, tannins, trypsin inhibitory substances, and dietary fiber are also present in millet. Phytochemicals such as dietary fiber and polyphenols are abundant in the millet seed coating, which is an edible part of the kernel [2].

The richness in dietary fibers, absence of gluten, and interesting protein and fat profiles are other properties that make these cereals very balanced food options that may help in the management of many disorders [11]. On the other hand, sorghum and pearl millet grains have a more variable nutritional and functional potential than major cereal grains [12]. Genetic factors, the growth environment, and the cultivation location all influence these variations [13]. Sorghum varieties that were resistant to biotic and abiotic stressors, for example, had higher levels of proanthocyanidins, 3-deoxyanthocyanidins, and flavan-4-ols on average than sensitive varieties [14].

An abundance of scientific studies report on the nutritional and bioactive properties of minor cereals. However, these studies generally concentrate on cultivars originating from America, Asia, and Europe, but very few African countries [15,16,17]. To the best of our knowledge, a comprehensive and comparative investigation of the chemical compositions of sorghum and pearl millet cultivars grown in Cameroon has not been reported. However, the characterization of local sorghum cultivars is an important step for breeding programs, nutrition policies, and the food and nutraceutical industries.

The goal of this research was to assess the nutritional and bioactive profiles of sorghum and pearl millet grain varieties that are regularly produced and consumed by locals. Moreover, the renewed interest in using sorghum and pearl millet to make value-added products for human nutrition and the lack of data on the chemical makeup of cultivars found in Cameroon’s Far-North region sustained this research.

## 2. Materials and Methods

### 2.1. Chemicals

Hexane, acetone, ethanol, methanol, HPLC-grade methanol, sodium carbonate, 2,20-azino-bis (3-ethylbenzothiazoline-6-sulfonic acid) diammonium salt (ABTS), 2,2-diphenyl1-picrylhydrazyl (DPPH), Folin–Ciocalteu reagent, gallic acid solution, hydrochloric acid, sodium nitrite, aluminum chloride, sodium hydroxide, iron (III) chloride, sulfosalicylic acid, phytic acid and potassium persulfate were purchased from Sigma Aldrich (Schnelldorf, Germany). The phenolic acid (gallic acid, vanillic acid, chlorogenic acid, caffeic acid, ferulic acid) and flavonoid (catechin, epicatechin, quercetin, and kaempferol) standards were purchased from Sigma Aldrich (Schnelldorf, Germany).

### 2.2. Samples Preparation

Sorghum (*white, yellow-pale, yellow*, and *red*) and pearl millet (*mouri*) grains were purchased from a local market in Maroua, the principal city of the Far-North region of Cameroon. The *gawane* cultivar of pearl millet was generously supplied by the Agricultural Research Institute of Development (IRAD)’s regional branch. The grains were cleaned and sorted to remove all dirt. After sun-drying, the grains were packed in polypropylene bags and stored at room temperature.

The grains were ground in a domestic grinder (Bosch TSM6A014R, 180 W) for 30 s, and the resulting flours (particle size 250 µm) were stored at −20 °C before analyses.

### 2.3. Characterization of Sorghum and Pearl Millet Flours

#### 2.3.1. Proximate Analysis

The moisture, lipid, protein, crude fiber, and ash contents of the flours were determined according to AOAC standard methods [11]. The carbohydrate content was determined by the difference between 100 and the total percentages of proteins, lipids, crude fibers, and ash [18].

#### 2.3.2. Determination of Mineral Content

The mineral contents of flours were determined using an atomic absorption spectrophotometer (3400 AAS Agilent Technologies). In brief, each flour sample (about 1 g) was burnt in a calcining furnace at 500 °C. Then, the ash was dissolved in 2.5 mL nitric acid (1 N) and filtrated. The filtrate was diluted using ultrapure water before analysis.

#### 2.3.3. Determination of Phytochemical Profile of Flours

The ultrasound-assisted extraction was performed by mixing 1 g of flour with 9 mL of 70% methanol (*v*/*v*) prepared with pure methanol and distilled water. The samples were vortexed for 5 min and then treated in an ultrasonic water bath (MRC Scientific Instruments) at 40 kHz and 30–35 °C for 30 min. Afterward, the samples were centrifuged (6000 rpm at 10 °C) for 10 min (Universal 320R Hettich Zentrifugen), followed by concentration to dryness of the supernatants with an AVC 2–18 concentrator (Christ, UK). All dried extracts were redissolved in 70% methanol (*v*/*v*) to reach a 10 mg/mL concentration for further analyses.

Total polyphenol content (TPC): The sorghum and millet flour TPC values were spectrophotometrically measured using the Folin–Ciocalteu method [19,20]. Briefly, 200 μL of the extract was mixed thoroughly with 15.8 mL of distilled water and 1 mL of Folin–Ciocalteau reagent. After 10 min, 3 mL of Na_2_CO_3_ 20% was added to the mixture. The resultant mixture was stored at room temperature in the dark for 60 min before being measured with a Biochrom Libra S22 spectrophotometer at 765 nm. The results were expressed as milligrams of gallic acid equivalents per gram of dry flour (mg GAE/g DW) using a calibration curve (0.1–0.5 mg/mL, R^2^ = 0.984).

Total flavonoid content (TFC): The aluminum chloride spectrophotometric method was used to determine the TFC values of sorghum and pearl millet flours [21]. Briefly, 0.25 mL of the extract was mixed with 2 mL of distilled water and 0.075 mL of 5% sodium nitrite (NaNO_2_). After 5 min, 0.15 mL of aluminum chloride (AlCl_3_) was added to the mixture. Six minutes later, 0.5 mL of sodium hydroxide (NaOH) 1 M was added to the mixture before being measured with a Biochrom Libra S22 spectrophotometer at 510 nm. The results were reported as milligrams of catechin equivalents per gram of dry flour (mg CE/g DW) using a calibration curve (0.1–0.5 mg/mL, R^2^ = 0.997).

Total 3-deoxyanthocyanidin content (TDC): The absorbance values of redissolved extracts were directly read at 480 nm, and the TDC was expressed as milligrams of 3-deoxyanthocyanidin per gram of flour (mg/g DW) using the molar extinction coefficient of luteolinidin, which is 13,800 M^−1^ cm^−1^ [22].

Total carotenoid content (TCC): Carotenoids were extracted as described above but using the hexane/acetone (3:2) mixture as the solvent. The absorbance values of redissolved dried extracts were directly read at 450 nm, and the TCC was expressed as milligrams per 100 g of dry flour (mg/100 g DW) using the molar extinction coefficient 2500 M^−1^ cm^−1^ [23].

Phytate content: The phytate content was determined according to the method described by Vaintraub and Lapteva [24], with slight modifications. Briefly, 0.5 g of either sorghum or pearl millet flour was extracted for 1 h at room temperature with 10 mL of 2.4% HCl. The mixture was centrifuged for 30 min at 3000 rpm at room temperature, and the supernatant was used to calculate the phytate. Three milliliters of the supernatant was mixed with one milliliter of Wade reagent (0.03% FeCl_3_ solution with 0.3% sulfosalicylic acid in distilled water) and centrifuged for 10 min at 3000 rpm at room temperature. Then, the absorbance was measured at 500 nm. A phytic acid standard curve (5–40 mg/mL, R^2^ = 0.980) was used to compute the phytate concentration, and the results were represented as phytic acids in milligrams per 100 g of dry weight (mg/100 g DW).

#### 2.3.4. Antioxidant Activity

DPPH scavenging method: The extracts’ antiradical activity was tested using the stable 2,2-diphenyl-1-picrylhydrazyl radical (DPPH) compound in accordance with the manufacturer’s instructions [25]. The ability of the extracts to bleach the free radical was used to examine their ability to scavenge free radicals. The blank absorbance was measured at 515 nm using a 3.9 mL DPPH solution 0.1 M (in methanol) and 0.100 mL methanol instead of the extract (*A*0). For the samples, 3.9 mL of 0.1 M DPPH solution was mixed with 0.100 mL of each extract, and afterward the mixtures were kept for 30 min at room temperature in the dark before the absorbances were recorded (*Af*). The inhibition percentage was calculated as follows:

(1) $$\%\;Inhibition=\frac{A0-Af}{A0}\times 100$$

ABTS scavenging method: For the ABTS scavenging activity, 2.85 mL of ABTS stock solution 7 mM (in ethanol, mixed with 2.45 mM potassium persulfate), having 1.10 absorbance (*A*0), was mixed with 0.15 mL of extract solution (*Af*). After 2 h of incubation in the dark, the solution’s absorbance was measured at 734 nm. The percentage of radical scavenging activity was estimated as follows:

(2) $$\%\;Inhibition=\frac{A0-Af}{A0}\times 100$$

#### 2.3.5. HPLC Analysis of Polyphenols

A chromatographic profile of sorghum and millet was created utilizing a Surveyor HPLC system (Finnigan Surveyor LC, Thermo Scientific, Waltham, MA, USA) using the following methodology for the preparation and separation of compounds, as described by Păcularu-Burada et al. [26]. The samples were suspended in 5 mL of 70% (*v*/*v*) methanol prior to HPLC separations. The mixtures were dissolved in an ultrasonic bath (MRC, Holon, Israel) for 45 min, and the sample supernatants were centrifuged at 6000 rpm and 4 °C for 10 min (Hettich Universal 320R, Tuttlingen, Germany) before being filtered through 0.22 m syringe filters (Merck, Darmstadt, Germany). Briefly, the elution system used was formed from 100% (*v*/*v*) methanol (solvent A) and 3% (*v*/*v*) formic acid (solvent B) and the separation was performed using a linear gradient: 0–20 min (91% B), 20–40 min (91–65% B), and 40–55 min (65–91% B). The analysis was conducted at detection wavelengths of 280 (for phenolic acids) and 320 nm (for flavonoids). The polyphenols were identified using the retention time for the commercially available standards as a guideline, and by comparison with the literature reviews.

### 2.4. Statistical Analysis

Unless otherwise stated, the analyses were performed in triplicate and the figures were reported as means ± standard deviation. Data were subjected to ANOVA and significant differences between means were revealed via post hoc Duncan’s multiple range test (*p* < 0.05). The principal component analysis (PCA) was carried out to gain an overview of the relationships among experimental data.

## 3. Results and Discussion

### 3.1. Proximate Chemical Composition of Sorghum and Pearl Millet Flours

The proximate physicochemical contents related to the main macronutrients (proteins, lipids, carbohydrates, and fibers), moisture, and ash in sorghum and pearl millet flours are shown in Table 1.

The moisture, protein, lipid, crude fiber, ash, and carbohydrate contents of the analyzed sorghum samples varied in the ranges of 8.51–9.33%, 19.62–23.78%, 2.74–3.32%, 2.56–4.70%, 1.15–1.59%, and 67.281–72.71%, respectively. As stated in Table 1, *white* sorghum was the most carbohydrate-rich cultivar with the highest moisture and ash contents. On the other hand, this cultivar had the lowest protein and fiber contents. The *yellow-pale* sorghum cultivar had the highest lipid concentration. The protein level of *yellow* sorghum was the highest, whereas its lipid content was the lowest. *Red* sorghum was determined to have the highest fiber content while having the lowest ash and carbohydrate levels. Our results are in agreement with other studies. Shegro et al. [27] and Udachan et al. [28] also reported differences in nutrient concentrations between different types of sorghum due to genetic differences. However, the concentrations identified in our study are comparable with those reported by these authors.

The carbohydrate and fiber content of pearl millet was significantly higher in the *gawane* cultivar (Table 1), whereas the protein, fat, and ash contents were significantly higher in the *mouri* cultivar (*p* < 0.05). By comparison, the pearl millet samples were higher in proteins (27.81–32.56%), lipids (5.11–5.36%), crude fibers (3.72–5.68%), and ash (2.24–2.77%) but lower in moisture (8.00–8.03%) and carbohydrates (55.57–59.09%) when compared to sorghum samples (Table 1). It is known that pearl millet has a better nutritional profile than sorghum and other major cereals, with the same findings being reported by other authors such as Ojo et al. [29].

The variations in the proximate compositions of the various samples may have been primarily due to genetic factors, since the studied cultivars were cultivated under similar climatic conditions [13]. Pearl millet cultivars, with low carbohydrate contents and high crude fiber contents, can be recommended to people suffering from metabolic syndrome, characterized by high glucose levels and hypercholesterolemia. In addition, considering the high levels of proteins and lipids, the studied pearl millet varieties are good candidates to fight protein-energy malnutrition in children.

### 3.2. The Mineral Content of Sorghum and Pearl Millet Flours

The mineral contents of the studied cereals are detailed in Table 2.

As shown in Table 2, *white* sorghum was the cultivar with the highest sodium and potassium levels, while having the lowest levels of magnesium and phosphorus. The *yellow-pale* sorghum cultivar had the highest calcium, magnesium, and phosphorus concentrations of all of the cultivars studied, but the lowest sodium concentration was found for the *yellow* sorghum cultivar. The lowest calcium and potassium concentrations were found in *red* sorghum. While *gawane* millet had a high sodium content, *mouri* pearl millet had the greatest calcium, magnesium, and phosphorus levels. No significant differences were observed between *gawane* and *mouri* pearl millet flours in the potassium concentrations (*p* > 0.05). By comparison, as already seen in Table 2, pearl millet presented a significantly higher calcium content than sorghum (*p* < 0.05).

The obtained results are comparable to data reported earlier for sorghum genotypes by authors such as Chavan et al. [30]. Other studies have reported pearl millet containing higher amounts of minerals compared to sorghum [31]. The cultivar with the lowest levels of copper, iron, and zinc was discovered to be *white* sorghum (Table 3). The copper and manganese concentrations in the *yellow-pale* sorghum were the highest of all the varieties evaluated. *Yellow* sorghum had the highest iron content but the lowest manganese content. *Red* sorghum also had a high copper content, with no significant difference from *yellow-pale* sorghum (*p* > 0.05). *Red* sorghum was also discovered to be the most zinc-rich cultivar. Although *gawane* pearl millet had the highest amounts of copper and zinc, *mouri* had the highest iron and manganese levels (Table 3).

As far as trace elements are concerned, copper, iron, and zinc contents were higher (*p* ˂ 0.05) in pearl millet cultivars compared to sorghum. Only manganese was more abundant in sorghum. Many studies reported pearl millet as a better source of minerals than the major cereals, especially iron [32]. The stated variations in the mineral contents of the studied cultivars can be explained by genetic factors and the soil composition [33]. Indeed, the soil characteristics are not homogeneous in the Far-North region of Cameroon [34]. Pearl millet grains can be used to prepare various nutrient-dense food products, effectively fighting mineral deficiency in children and women.

### 3.3. Phytochemicals Profile of Sorghum and Pearl Millet Flours

The phytochemical composition of the extracts derived from sorghum and pearl millet was determined. Table 4 shows the concentrations of TFC, TPC, TDC, TCC, and phytates.

The phytochemical profile showed significant variations among the studied cultivars. According to Table 4, *red* sorghum was the richest cultivar in total polyphenols, total flavonoids, and total 3-deoxyanthocyanidin. However, the lowest phytate contents were noticed in *red* sorghum. One of the elements determining the content and profile of flavonoids is the color of the sorghum grains [35,36]. Sorghum grains contain flavonoids in the form of 3-deoxyanthocyanidins [12,32]. Polyphenols are the main bioactive compounds of sorghum and are present in all cultivars of this cereal crop [37]. *White* sorghum was the richest cultivar in total carotenoids. A recent study conducted in Poland on sorghum grains also revealed the white genotype to have the highest content in carotenoids [37]. These findings suggest that the pigmentation of the external coat of sorghum cultivars is not an indicator of the abundance of carotenoids, as it is the case with many fruits and vegetables.

The *mouri* pearl millet was the richest source of total polyphenols and TDC, while *gawane* showed the highest phytate content. In terms of the TFC and TCC, no significant differences were observed between the two cultivars of pearl millet (*p* > 0.05).

In the present study, the analyzed compounds were globally found in higher amounts in sorghum varieties compared to pearl millet. However, polyphenols and phytates are also known as antinutritional factors since they form insoluble complexes with minerals such as iron, zinc, and calcium, reducing their bioavailability [38].

### 3.4. Antioxidant Activity of Sorghum and Pearl Millet Flours

The radical scavenging activities of sorghum and pearl millet flour varieties are presented in Table 5, with DPPH and ABTS being used as free radicals.

The values from Table 5 reveal that the sorghum varieties were high in DPPH antioxidant activity compared to the pearl millet cultivars. The highest antioxidant activity was for *yellow-pale* sorghum (93.14%), followed by *red* sorghum (86.43%). Punia et al. [39] found *red* sorghum to exhibit the highest antioxidant activity among five varieties cultivated in India. Another recent study carried out in the Mediterranean zone also identified *red* sorghum to have high antioxidant potential [40]. However, it can be noticed that no sorghum variety with a yellowish pericarp was used in these two previous studies.

The antioxidant activity levels of pearl millet flours tested against DPPH radical were more than two-fold higher than the result obtained by Gull et al. (31.80%) in a pearl millet variety grown in India [41]. In addition to genetic factors, the observed difference may be attributed to the local harsh and stressful climatic conditions, which could have boosted the synthesis of antioxidant phytochemicals.

In terms of ABTS, no significant differences were observed in the inhibition activity levels of the sorghum and pearl millet flours (*p* > 0.05). The antioxidant properties of cereal grains are attributed to their polyphenols and flavonoids, which act as free radical scavenging agents and protect against oxidative stress within the human body [42].

### 3.5. High-Performance Liquid Chromatography Analysis

**Figure 1:**
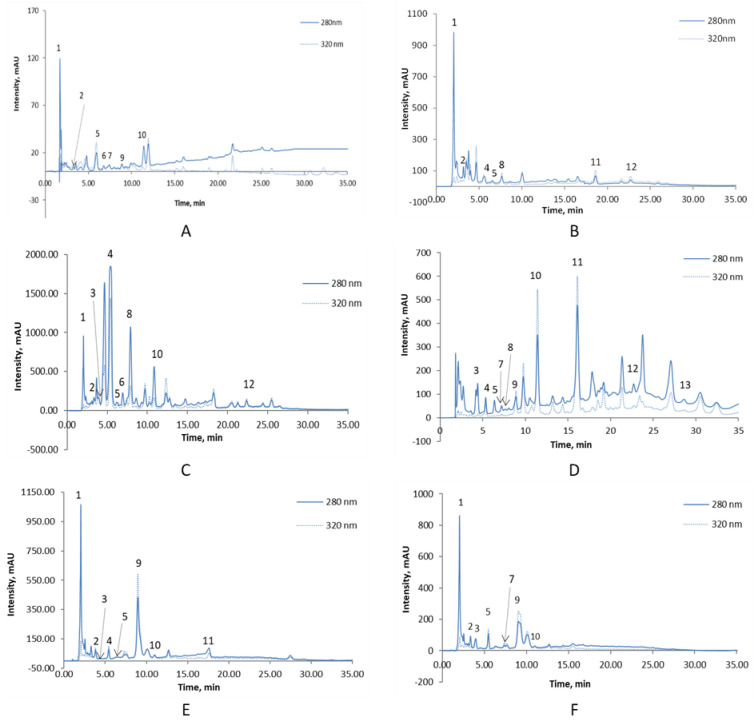
Chromatographic profile of sorghum and pearl millet phenols detected at 280 nm and 320 nm: peak 1—gallic acid; peak 2—protocatechuic acid; peak 3—catechin; peak 4—vanillic acid; peak 5—chlorogenic acid; peak 6—epicatechin; peak 7—caffeic acid; peak 8—syringic acid; peak 9—p coumaric acid; peak 10—ferulic acid; peak 11—sinapic acid; peak 12—quercetin; 13—kaempferol; (**A**)—*white* sorghum; (**B**)—*yellow-pale* sorghum; (**C**)—*yellow* sorghum; (**D**)—*red* sorghum; (**E**)—*gawane* millet’ (**F**)—*mouri* millet.

Figure 1 illustrates the chromatographic profile of sorghum and millet flour varieties. At 320 nm, 13 compounds were identified: caffeic acid, chlorogenic acid, ferulic acid, gallic acid, p coumaric acid, protocatechuic acid, sinapic acid, syringic acid, and vanillic acid. Catechin, epicatechin, quercetin, and kaempferol were the flavonoid compounds that were separated.

Table 6 shows the concentrations of bioactive compounds identified in sorghum and pearl millet varieties.

The polyphenolic extracts of sorghum and pearl millet revealed different concentrations of each bioactive compound in the analysis. It can be seen from Figure 1 and Table 6 that the main compounds identified were gallic acid, chlorogenic acid, and ferulic acid. Chlorogenic acid, caffeic acid, p-coumaric acid, ferulic acid, and sinapic acid were the hydroxycinnamic acids measured (Figure 1), with the different levels among the genotypes (Table 6) attributed to the cultivars and the growth conditions and environment they were exposed to [39]. Among them, the most frequent phenolic acids identified were chlorogenic acid and ferulic acid. Ferulic acid was higher in *red* and *yellow* sorghum while chlorogenic acid was higher in *yellow* sorghum and *mouri* pearl millet. Similar results were obtained in previous studies [40,43].

The hydroxybenzoic acids identified were gallic acid, protocatechuic acid, vanillic acid, and syringic acid (Figure 1). Vanillic acid was most prominent in *yellow* sorghum and was lacking in *white* sorghum.

As indicated in Table 6, *red* sorghum had a more considerable phenolic compound diversity than the other samples, followed by *gawane* pearl millet. In their studies, Ghinea et al. also identified that sorghum bicolor grains exhibited a high diversity of compounds such as caffeic acid, chlorogenic acid, epicatechin, p-coumaric acid, daidzein, rutin, hyperoside, quercetin, naringenin, and genistein [44]. Another study by Hong et al. found a variety of polyphenolic compounds isolated from sorghum extracts, including caffeic acid, coumaric acid, ellagic acid, ferulic acid, gallic acid, sinapic acid, syringic acid, vanillic acid, apigenin, catechin, chrysin, eriodictyol, luteolin, naringenin, and quercetin [45].

### 3.6. Principal Components Analysis (PCA) of Experimental Data

**Figure 2:**
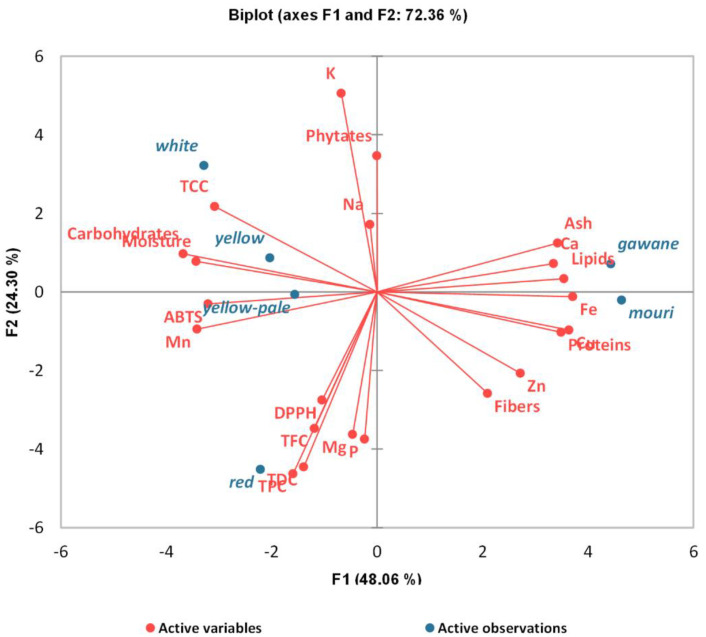
Principal components analysis of the experimental data.

A principal component analysis was carried out to determine and visualize the relationships between the different sorghum and pearl millet cultivars and the studied parameters. The eigenvalues were 10.57, 5.35, 3.13, 1.71, and 1.24 for factors F1 to F5, respectively. The first two principal components or factors (F1 and F2) together explained 72.96% of the total original variance in the data set. A biplot projection of the observations (cultivars) and measured variables on the plane defined by F1 and F2 is shown in Figure 2.

The first principal component F1, which explained 48.06% of the total experimental variability, aggregated all of the proximate analysis parameters, trace elements, and calcium. The location of *gawane* and *mouri* pearl millet close to proteins, lipids, and trace elements such as copper (Cu), iron (Fe), and zinc (Zn) clearly indicates that these cultivars are better sources of nutrients compared to sorghum. The tight positive correlation between the proteins and the above-mentioned trace elements may be due to the mineral-binding ability of some amino acid side chains. Additionally, our findings suggest a possible competitive binding process among divalent cations favorable to calcium, copper, iron, and zinc. *Yellow-pale* and *yellow* sorghum are characterized by high carbohydrate, carotenoid, and manganese (Mn) contents, which have a strong negative correlation with the majority of nutrients. The carotenoids may be the main contributors to the ABTS antioxidant activity observed in the studied samples.

The second principal component F2, which explained 24.30% of the total experimental variability, clustered phytochemicals with antioxidant properties (TDC, TFC, TPC), phytates, and macro-elements such as magnesium (Mg), phosphorus (P), potassium (K), and sodium (Na). Phytates negatively correlated with other phytochemicals. There is a strong positive correlation between the grouped phytochemicals, which are synthesized in the context of a physiological adaptation of the plant to environmental stress. Additionally, it can be deduced by the position of *red* sorghum being close to TDC, TFC, TPC, and DPPH that it had the best antioxidant profile. The independent relationship between trace elements and phytochemicals such as polyphenols and phytates is a paradoxal finding. In fact, these two compounds are known to be divalent cations chelators [46].

The principal component analysis clearly segregated pearl millet cultivars (especially *mouri*) and *red* sorghum as the major sources of nutrients and bioactive compounds, respectively. This implies that recommending their consumption will depend on the type of nutritional challenges faced. *Mouri* pearl millet seems to be suitable for addressing protein-energy and micronutrients deficiencies, while *red* sorghum could be useful in managing non-communicable diseases.

## 4. Conclusions

This study analyzed the chemical composition and antioxidant profile of four sorghum and two pearl millet cultivars grown and consumed in the Far-North region of Cameroon. The physicochemical and mineral analyses revealed a global nutritional superiority of pearl millet cultivars, especially *mouri*, which exhibited high amounts of proteins and trace elements. The phytochemicals and antioxidant activity were noticeably higher in colored cultivars of sorghum (*red* and *yellow*). *Red* sorghum had a more considerable phenolic compound diversity than the other samples. The most abundant phenolic component was gallic acid, while ferulic acid was the most dominant. The studied cereal crops can be considered health-promoting tools for local populations, and their consumption should be encouraged to tackle nutrient deficiencies and non-communicable diseases. However, further investigations are required to identify adequate processing methods that best preserve the nutritional and functional properties mentioned above.

## Figures and Tables

**Table 1:** Proximate chemical composition of sorghum and pearl millet flours (g/100 g DW).

Samples	Moisture (%)	Proteins	Lipids	Fibers	Ash	Carbohydrates
**Sorghum Varieties**						
*white*	9.33 ± 0.01 ^a^	19.62 ± 0.01 ^a^	3.49 ± 0.02 ^a^	2.56 ± 0.02 ^a^	1.59 ± 0.01 ^a^	72.71± 0.02 ^a^
*yellow-pale*	8.51± 0.01 ^b^	23.21 ± 0.01 ^b^	3.62 ± 0.01 ^b^	3.39 ± 0.01 ^b^	1.44 ± 0.01 ^b^	68.31 ± 0.04 ^b^
*yellow*	8.63 ± 0.01 ^c^	23.78 ± 0.01 ^c^	2.74 ± 0.01 ^c^	3.79 ± 0.03 ^c^	1.21 ± 0.01 ^c^	68.45 ± 0.02 ^c^
*red*	8.85 ± 0.01 ^d^	23.51 ± 0.01 ^d^	3.33 ± 0.01 ^d^	4.70 ± 0.03 ^d^	1.15 ± 0.01 ^d^	67.28 ± 0.02 ^d^
**Pearl Millet Varieties**						
*gawane*	8.00 ± 0.01 ^e^	27.85 ± 0.01 ^e^	5.11 ± 0.01 ^e^	5.68 ± 0.03 ^e^	2.24 ± 0.01 ^e^	59.09 ± 0.01 ^e^
*mouri*	8.03 ± 0.01 ^f^	32.56 ± 0.02 ^f^	5.36 ± 0.01 ^f^	3.72 ± 0.05 ^c^	2.77 ± 0.01 ^f^	55.57 ± 0.04 ^f^

DW: dry weight of flour. Mean values in the same column with different superscript letters are significantly different (*p* < 0.05).

**Table 2:** Macro-element (mg/100 g DW) and trace element contents of sorghum and pearl millet flours (mg/100 g DW).

Samples	Ca	Na	K	Mg	P
**Sorghum Varieties**					
*white*	11.20 ± 0.08 ^a^	4.55 ± 0.03 ^c^	328.70 ± 0.89 ^d^	130.47 ± 0.13 ^a^	256.74 ± 1.04 ^a^
*yellow-pale*	12.91 ± 0.09 ^b^	3.98 ± 0.13 ^a^	313.39 ± 8.31 ^c^	149.11 ± 0.46 ^b^	311.37 ± 0.13 ^b^
*yellow*	11.90 ± 0.01 ^c^	3.94 ± 0.01 ^a^	314.34 ± 1.10 ^c^	139.35 ± 0.03 ^c^	279.73 ± 0.76 ^c^
*red*	10.81 ± 0.03 ^d^	4.21 ± 0.01 ^b^	278.68 ± 0.21 ^a^	145.69 ± 0.63 ^d^	301.77 ± 0.35 ^d^
**Pearl Millet Varieties**					
*gawane*	13.66 ± 0.09 ^e^	4.48 ± 0.05 ^c^	302.52 ± 1.36 ^b^	132.47 ± 0.21 ^e^	266.03 ± 0.90 ^e^
*mouri*	15.67 c ± 0.30 ^f^	3.96 ± 0.03 ^a^	307.06 ± 1.92 ^bc^	142.48 ± 0.40 ^f^	292.66 ± 0.12 ^f^

DW: dry weight of flour. Mean values in the same column with different superscript letters are significantly different (*p* < 0.05).

**Table 3:** Macro-element (mg/100 g DW) and trace element contents of sorghum and pearl millet flours (mg/100 g DW).

Samples	Cu	Fe	Mn	Zn	Samples
**Sorghum Varieties**					
*White*	0.12 ± 0.07 ^a^	2.75 ± 0.15 ^a^	1.48 ± 0.05 ^a^	1.34 ± 0.01 ^a^	*White*
*yellow-pale*	0.25 ± 0.07 ^c^	3.15 ± 0.08 ^b^	1.69 ± 0.01 ^b^	1.71 ± 0.01 ^b^	*yellow-pale*
*yellow*	0.21 ± 0.08 ^b^	3.28 ± 0.07 ^c^	1.40 ± 0.02 ^c^	1.38 ± 0.03 ^c^	*yellow*
*red*	0.32 ± 0.08 ^c^	3.07 ± 0.01 ^d^	1.56 ± 0.01 ^d^	2.13 ± 0.06 ^d^	*red*
**Pearl Millet Varieties**					
*Gawane*	0.69 ± 0.03 ^e^	4.45 ± 0.01 ^e^	0.54 ± 0.01 ^e^	2.78 ± 0.02 ^e^	*Gawane*
*Mouri*	0.59 ± 0.06 ^d^	4.92 ± 0.04 ^f^	0.92 ± 0.01 ^f^	1.97± 0.01 ^f^	*Mouri*

DW: dry weight of flour. Mean values in the same column with different superscript letters are significantly different (*p* < 0.05).

**Table 4:** Phytochemicals content of sorghum and pearl millet flours.

Samples	TPC (mg GAE/g DE)	TFC (mg CE/g DE)	TDC (mg/g DE)	TCC (mg/100 g DE)	Phytates (mg/100 g DW)
**Sorghum Varieties**					
*white*	22.48 ± 0.75 ^a^	7.14 ± 0.34 ^a^	1.60 ± 0.03 ^a^	0.99 ± 0.10 ^a^	330.44 ± 19.59 ^a^
*yellow-pale*	33.96 ± 0.80 ^b^	5.18 ± 0.64 ^b^	1.04 ± 0.05 ^b^	0.94 ± 0.02 ^a^	391.00 ± 18.95 ^b^
*yellow*	21.91 ± 0.93 ^a^	19.97 ± 0.52 ^c^	1.20 ± 0.02 ^b^	0.74 ± 0.02 ^b^	389.91 ± 24.57 ^b^
*red*	82.22 ± 3.29 ^c^	23.82 ± 1.27 ^d^	9.06 ± 0.32 ^c^	0.66 ± 0.03 ^c^	223.33 ± 12.24 ^c^
**Pearl Millet Varieties**					
*gawane*	17.36 ± 0.44 ^a^	9.23 ± 0.10 ^e^	0.74 ± 0.01 ^d^	0.52 ± 0.03 ^d^	384.93 ± 18.28 ^b^
*mouri*	19.15 ± 0.56 ^a^	8.85 ± 0.06 ^e^	1.01 ± 0.05 ^b^	0.53 ± 0.01 ^d^	273.60 ± 15.54 ^d^

TPC: Total Polyphenols Content; TFC: Total Flavonoids Content; TDC: Total 3-Deoxyanthocyanidin Content; TCC: Total Carotenoids Content; GAE: Gallic Acid Equivalent; CE: Catechin Equivalent; DW: dry weight of flour; DE: dry weight of extract; Mean values in the same column with different superscript letters are significantly different (*p* < 0.05).

**Table 5:** Antioxidant activity of sorghum and millet extracts.

Samples	DPPH (%)	ABTS (%)
**Sorghum Cultivars**		
*white*	73.18 ± 5.26 ^b^	95.02 ± 5.51 ^a^
*yellow-pale*	93.14 ± 4.46 ^c^	92.65 ± 6.76 ^a^
*yellow*	64.09 ± 3.29 ^a^	98.14 ± 5.48 ^a^
*red*	86.43 ± 5.03 ^c^	95.77 ± 3.97 ^a^
**Pearl Millet Cultivars**		
*gawane*	70.55 ± 3.62 ^ab^	90.64 ± 4.48 ^a^
*mouri*	73.27 ± 5.36 ^b^	89.24 ± 1.64 ^a^

Mean values in the same column with different superscript letters are significantly different (*p* < 0.05).

**Table 6:** The concentrations of compounds detected in sorghum and pearl millet cultivars.

Bioactive Compounds Identified (μg/mL)	Sorghum Cultivars			Pearl Millet Cultivars		
	*White*	*Yellow-Pale*	*Yellow*	*Red*	*Gawane*	*Mouri*
Gallic Acid	115.11 ± 2.00 ^c^	170.01 ± 0.80 ^b^	104.73 ± 0.90 ^d^	ND	185.79 ± 0.70 ^a^	165.59 ± 0.10 ^b^
Catechin	ND	ND	NQ	87.41 ± 0.90 ^a^	12.65 ± 0.60 ^c^	75.73 ± 0.30 ^b^
Vanillic Acid	ND	21.34 ± 0.7	548.65 ± 1.40 ^b^	18.24 ± 0.04 ^a^	20.27 ± 0.20 ^b^	ND
Chlorogenic Acid	10.11 ± 0.10 ^f^	22.37 ± 0.11 ^d^	59.36 ± 0.09 ^a^	32.71 ± 0.08 ^c^	19.06 ± 0.07 ^e^	34.61 ± 0.12 ^b^
Epicatechin	ND	ND	114.80 ± 2.69 ^a^	ND	ND	ND
Caffeic Acid	7.42 ± 0.04 ^a^	ND	ND	4.72 ± 0.01 ^b^	ND	4.32 ± 0.05 ^c^
Ferulic Acid	2.73 ± 0.22 ^d^	5.11 ± 0.07 ^c^	55.96 ± 0.17 ^a^	34.16 ± 0.49 ^b^	NQ	NQ
Quercetin	ND	ND	ND	53.85 ± 0.74 ^a^	ND	ND
Kaempferol	ND	ND	ND	11.49 ± 0.10 ^a^	ND	ND

ND: not detected; NQ: not quantified. Mean values in the same column with different superscript letters are significantly different (*p* < 0.05).

## Data Availability

Data is contained within the article.
